# Supplementary material for: Pheromone-based communication influences the production of somatic extracellular vesicles in C. elegans
Source: Nat Commun. 2024 Mar 28;15:2715. doi: 10.1038/s41467-024-47016-x (PMC10978837; doi:10.1038/s41467-024-47016-x)
Supplement: Supplementary file 1 — Supplementary Information [file 41467_2024_47016_MOESM1_ESM.pdf]

Supplementary Information

Pheromone-based communication influences the production of somatic extracellular vesicles in *C. elegans*

Agata Szczepańska[1#], Katarzyna Olek[2#], Klaudia Kołodziejska[1], Jingfang Yu[3], Abdulrahman Tudu Ibrahim[1,4], Laura Adamkiewicz[1], Frank C. Schroeder[3], Wojciech Pokrzywa[2*], Michał Turek[1*]

Affiliations:

[1]*Laboratory of Animal Molecular Physiology, Institute of Biochemistry and Biophysics, Polish Academy of Sciences, Warsaw, Poland*

[2]*Laboratory of Protein Metabolism, International Institute of Molecular and Cell Biology in Warsaw, Poland.*

[3]*Boyce Thompson Institute and Department of Chemistry and Chemical Biology, Cornell University, Ithaca, NY 14853, USA*

[4]*Faculty of Chemistry, Warsaw University of Technology, Warsaw, Poland*

[#] These authors contributed equally

* Co-corresponding authors

Correspondence should be directed to:

MT: m.turek@ibb.waw.pl

WP: wpokrzywa@iimcb.gov.pl

The **Supplementary Information** contains 7 figures, 2 tables, 3 spreadsheets, and a reporting summary.

Supplementary Figures

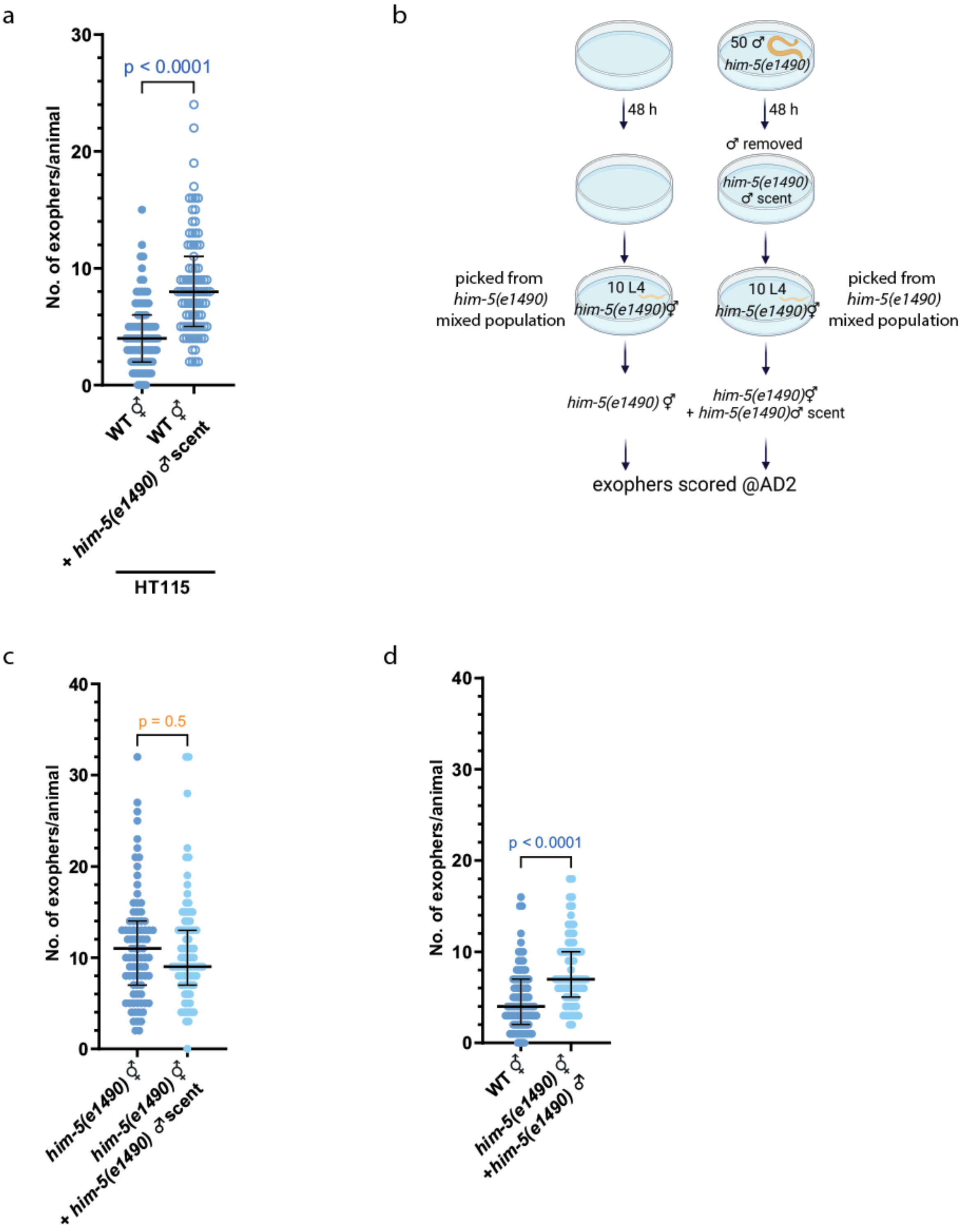

Supplementary Figure 1. Exposing hermaphrodites to males or their scent throughout the entire duration of the experiment does not further elevate exopher production.

a Growing hermaphrodites on male-conditioned plates increases exophergenesis levels also when worms were grown on HT115 *E. coli* strain. n = 79 and 87 worms (for respective columns), N = 3 independent experiments.

b Schematic representation of the experimental setup for Supplementary Fig. 1c. Created with BioRender.com.

c Exposing hermaphrodites to male secretome after the L4 stage does not further increase exopher production. n = 83 worms, N = 3 independent experiments.

d Co-culturing *him-5* hermaphrodites with males increases exophergenesis. n = 90 and 72 worms (for respective columns), N = 3 independent experiments.

Data information: Data are presented as median with interquartile range; not significant p values ($p > 0.05$) are in orange color, significant p values ($p < 0.05$) are in blue color; two-tailed Mann-Whitney test. Source data are provided as a Source Data file.

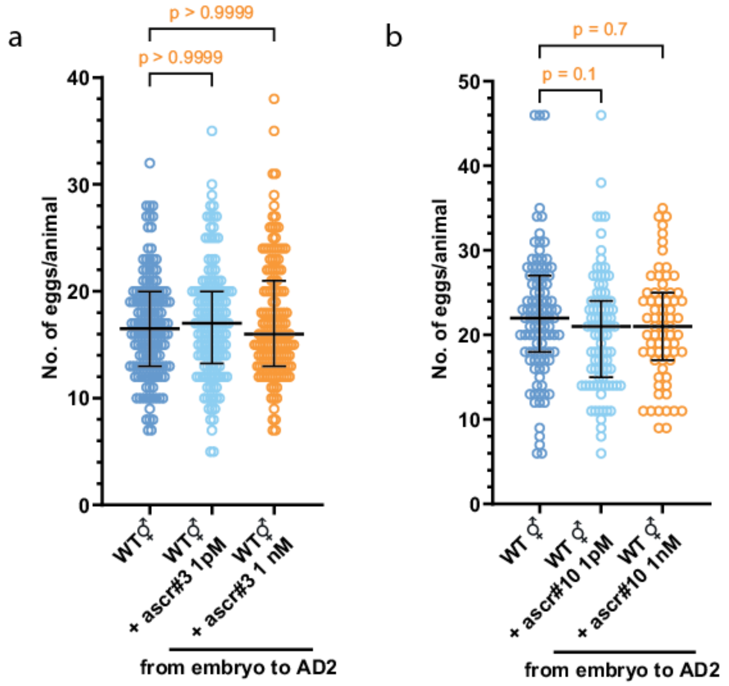

Supplementary Figure 2. ascr#3 and ascr#10 do not influence embryo retention in hermaphrodites.

a ascr#3 does not affect embryo retention in hermaphrodites. n = 140 worms, N = 5 independent experiments.

b ascr#10 does not affect embryo retention in hermaphrodites. n = 101, 93, and 69 worms (for respective columns), N = 4 independent experiments.

Data information: Data are presented as median with interquartile range; not significant p values ($p > 0.05$) are in orange color, Kruskal-Wallis test with Dunn's multiple comparisons test. Source data are provided as a Source Data file.

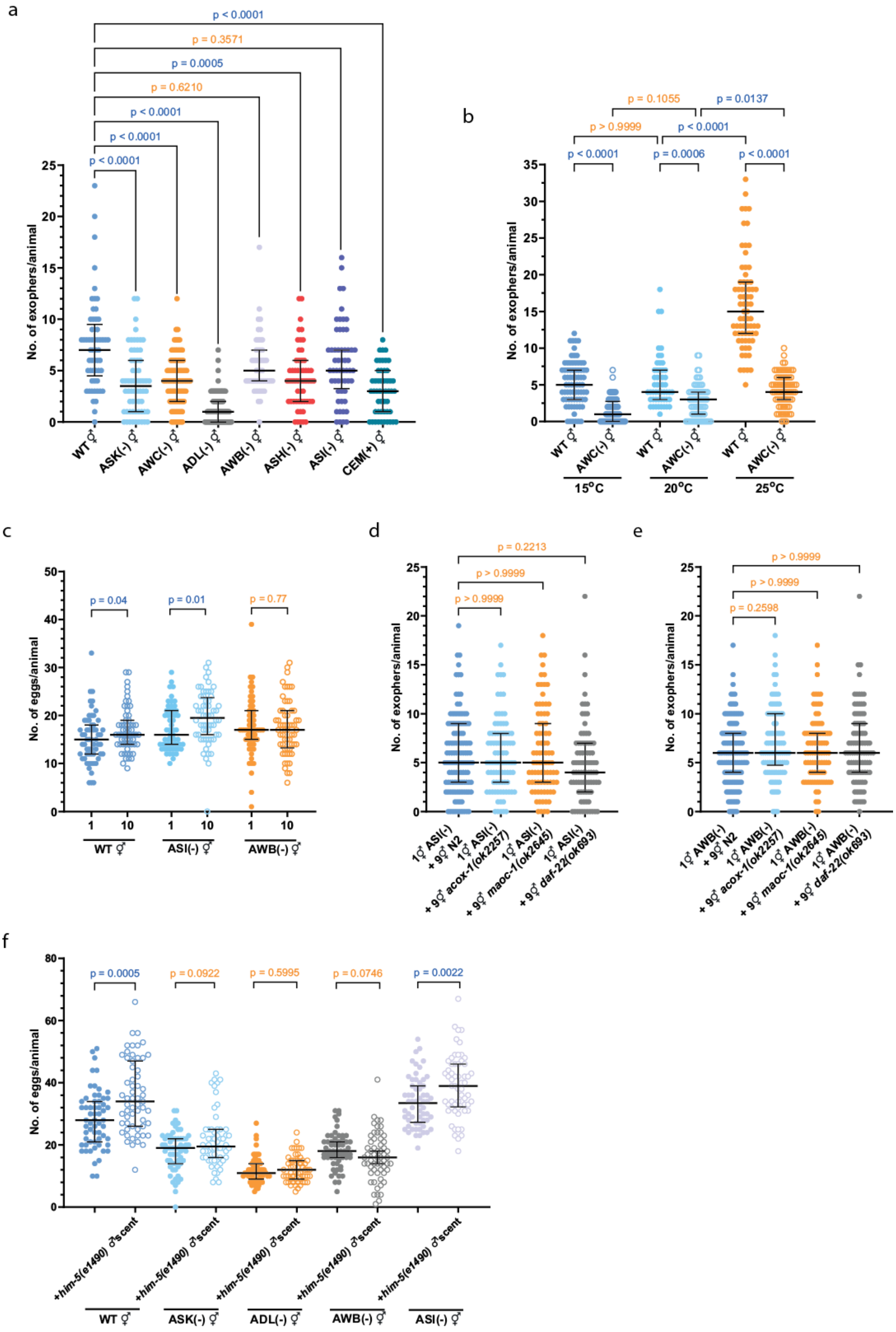
a
p < 0.0001
p = 0.3571
p = 0.0005
p = 0.6210
p < 0.0001
p < 0.0001
p < 0.0001
No. of exophers/animal
WT ⚥
ASK(-) ⚥
AWC(-) ⚥
ADL(-) ⚥
AWB(-) ⚥
ASH(-) ⚥
ASI(-) ⚥
CEM(+) ⚥
b
p = 0.1055
p = 0.0137
p > 0.9999
p < 0.0001
p < 0.0001
p = 0.0006
p < 0.0001
No. of exophers/animal
WT ⚥
AWC(-) ⚥
15°C
20°C
25°C
c
p = 0.04
p = 0.01
p = 0.77
No. of eggs/animal
WT ⚥
ASI(-) ⚥
AWB(-) ⚥
d
p = 0.2213
p > 0.9999
p > 0.9999
No. of exophers/animal
1⚥ ASI(-) + 9⚥ N2
1⚥ ASI(-) + 9⚥ acox-1(ok2257)
1⚥ ASI(-) + 9⚥ maoc-1(ok2645)
1⚥ ASI(-) + 9⚥ daf-22(ok693)
e
p > 0.9999
p > 0.9999
p = 0.2598
No. of exophers/animal
1⚥ AWB(-) + 9⚥ N2
1⚥ AWB(-) + 9⚥ acox-1(ok2257)
1⚥ AWB(-) + 9⚥ maoc-1(ok2645)
1⚥ AWB(-) + 9⚥ daf-22(ok693)
f
p = 0.0005
p = 0.0922
p = 0.5995
p = 0.0746
p = 0.0022
No. of eggs/animal
+him-5(e1490) ♂scent
WT ⚥
ASK(-) ⚥
ADL(-) ⚥
AWB(-) ⚥
ASI(-) ⚥

Supplementary Figure 3. Influence of pheromones released by ascaroside biosynthesis mutants on hermaphrodites with genetically ablated sensory neurons.

a Genetic ablation of ASK, AWC, ADL, or ASH neurons reduces exopher production in hermaphrodites grown as solitary animals. Solitary hermaphrodites with male-specific CEM neurons produce fewer exophers than wild-type worms. Data for solitary animals were extracted from Fig. 4e and presented separately to enhance accessibility of the statistical analysis. n = 57, 62, 84, 88, 49, 55, 56, and 50 worms (for respective columns), N = 3 independent experiments.

b AWC neurons regulate the temperature-dependent increase in muscle exopher production. n = 71, 73, 52, 74, 75, and 92 worms (for respective columns), N = 3 independent experiments.

c *In utero* embryo number for wild-type worms and ASI and AWB ablation mutants grown as solitary animals or in ten-hermaphrodite population. n = 60, 60, 55, 60, 60, and 60 worms (for respective columns), N = 3 independent experiments.

d - e Worms with ablated ASI and AWB neurons grown together with ascaroside biosynthesis mutants do not exhibit alterations in produced exopher numbers. (d) n = 117, 87, 83, and 83 worms (for respective columns), (e) n = 120, 90, 87, and 90 worms (for respective columns), N = 3 independent experiments.

f Removal of ASK, AWB, or ADL neurons prevented the increase in the number of embryos *in utero* after exposing hermaphrodites to male pheromones. n = 60, 59, 60, 60, 60, 55, 60, 59, 60, and 60 worms (for respective columns), N = 3 independent experiments.

Data information: Data are presented as median with interquartile range; n represents the number of worms; (a - b, d -e) Kruskal-Wallis test with Dunn's multiple comparisons test, (c, f) two-tailed Mann-Whitney test. Source data are provided as a Source Data file.

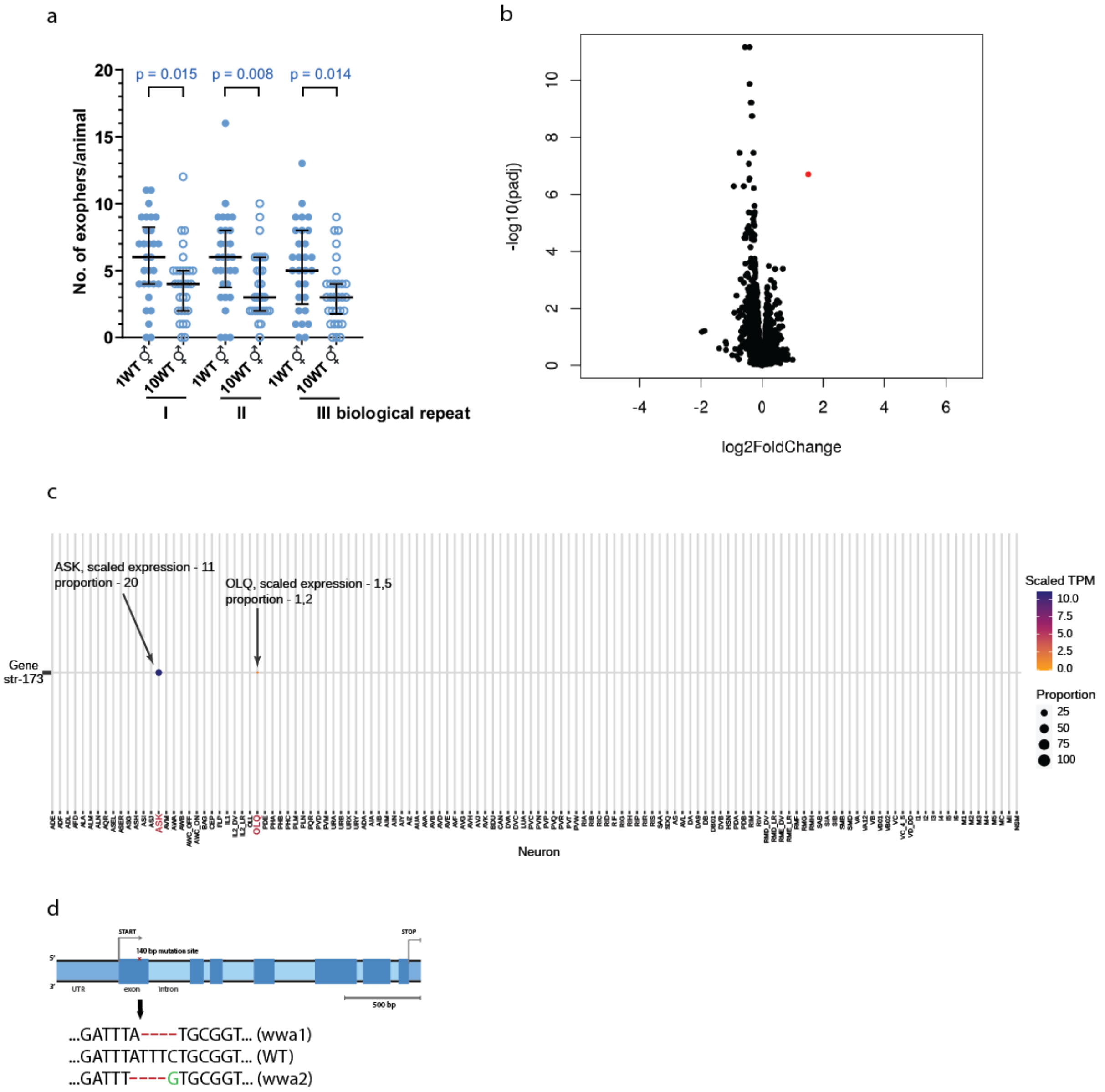

Supplementary Figure 4. Comparison of transcriptomes of worms grown at different population densities.

a Number of exophers produced by worms that were used for RNAseq analysis. n = 30, 30, 30, 30, 29, and 30 worms (for respective columns), N = 3 independent experiments.

b Transcriptomes of worms grown as single animals or at a density of ten hermaphrodites per plate did not differ dramatically. One transcript with a highly significant change is marked as a red dot.

c Single-cell RNA-seq data from CeNGENApp[39] show *str-173* strong expression in ASK neurons and weak expression in OLQ neurons. The circle diameter represents the proportion of neurons in each cluster that express the *str-173* gene.

d Localization of *str-173* mutations in the gene.

Data information: Data are presented as median with interquartile range; significant p values ($p < 0.05$) are in blue color; (a) two-tailed Mann-Whitney test. Source data are provided as a Source Data file.

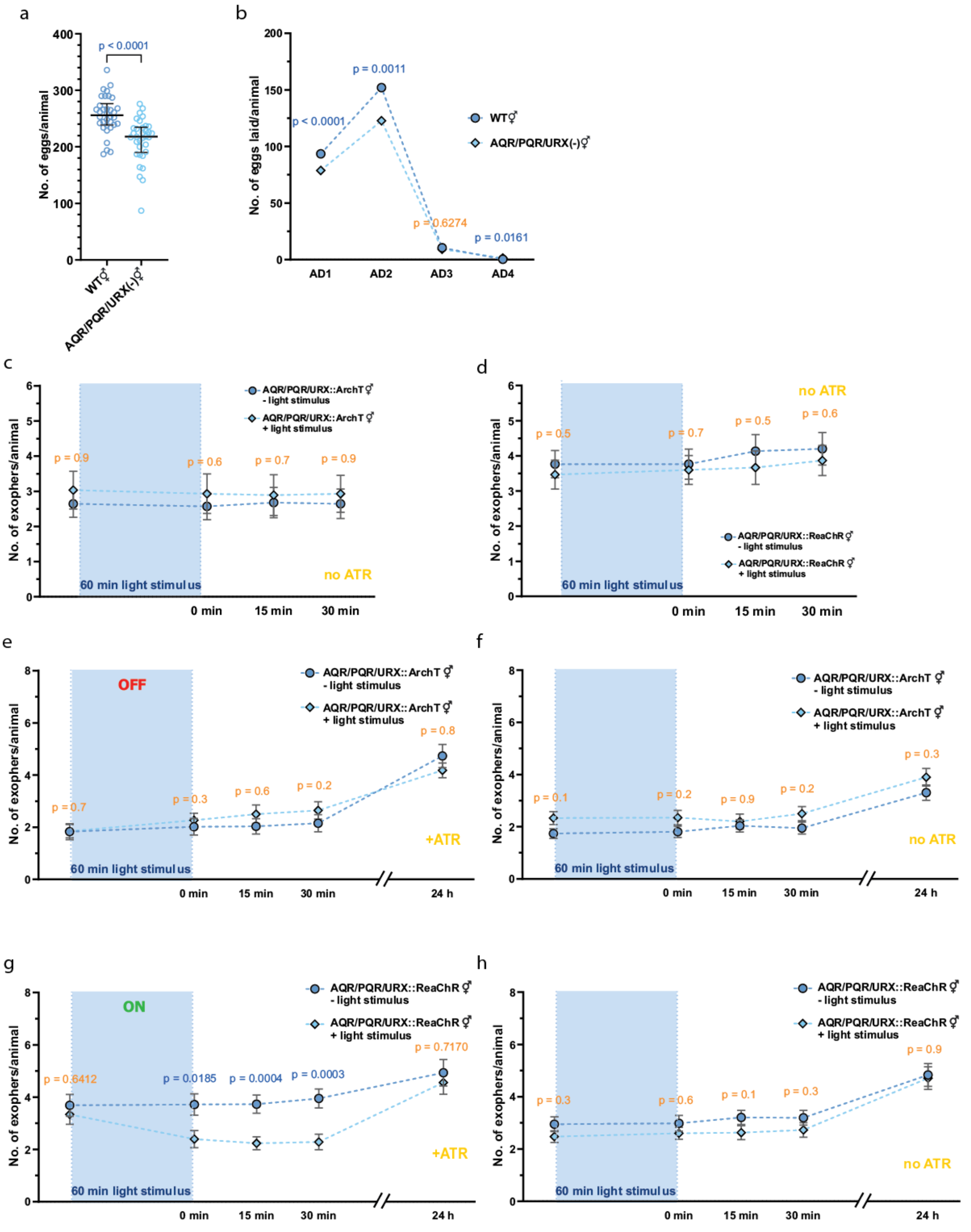

Supplementary Figure 5. ReaChR-based activation but not ArchT-based inactivation at the adult day 1 (AD1) stage influences exopher production in hermaphrodites.

a - b Worms with the genetic ablation of pseudocoelom-exposed AQR, PQR, and URX neurons have lower brood size than the wild type. n = 34 worms, N = 3 independent experiments.

c - d Control experiment without all-trans retinal (ATR) for ArchT-mediated inactivation and ReaChR-mediated activation of AQR, PQR, and URX neurons. (c) n = 23 worms (-light stimulus), and 28 worms (+ light stimulus), N = 3 independent experiments; (d)) n = 30 worms, N = 3 independent experiments.

e – f ArchT-mediated inactivation of AQR, PQR, and URX neurons at the adult day 1 (AD1) stage does not modulate exopher production. (e) n = 59 worms (-light stimulus), and 60 worms (+ light stimulus), (f) n = 57 worms (-light stimulus), and 60 worms (+ light stimulus), N = 6 independent experiments.

g – h ReaChR-mediated activation of AQR, PQR, and URX neurons at the adult day 1 (AD1) stage significantly decreases exopher production. (g) n = 61 worms (-light stimulus), and 60 worms (+ light stimulus), (h) n = 62 worms (-light stimulus), and 59 worms (+ light stimulus), N = 6 independent experiments.

Data information: +ATR means “with all-trans-retinal”; no ATR means “without all-trans-retinal”. Data are presented as median with interquartile range (a), mean (b), or mean with SEM (c – h); not significant p values ($p > 0.05$) are in orange color, significant p values ($p < 0.05$) are in blue color; two-tailed Mann-Whitney test. Source data are provided as a Source Data file.

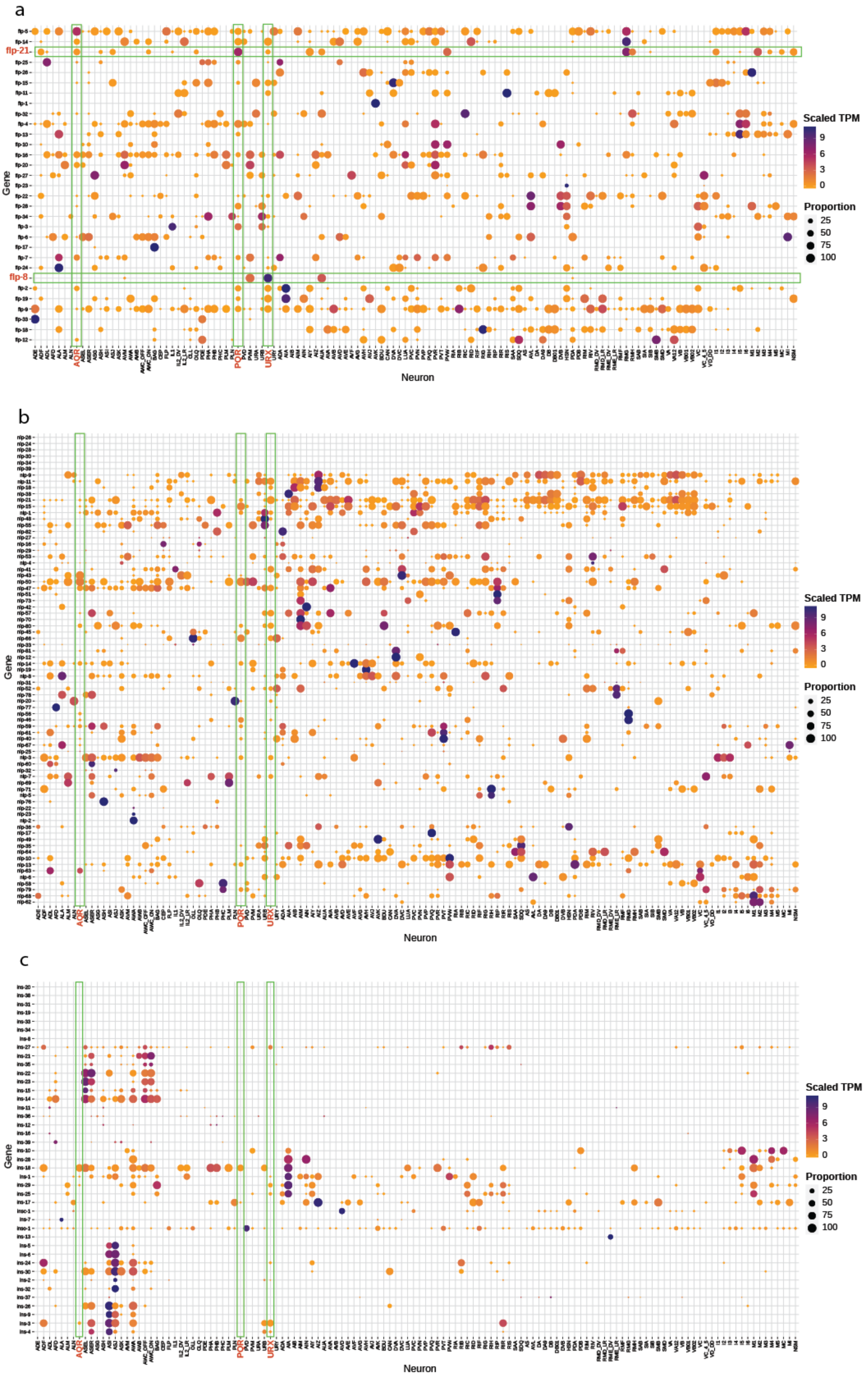
a
flp-21
flp-8
AQR
PQR
URX
Gene
Neuron
Scaled TPM
9
6
3
0
Proportion
25
50
75
100
b
AQR
PQR
URX
Gene
Neuron
Scaled TPM
9
6
3
0
Proportion
25
50
75
100
c
AQR
PQR
URX
Gene
Neuron
Scaled TPM
9
6
3
0
Proportion
25
50
75
100

Supplementary Figure 6. FLP-8 and FLP-21 are expressed in URX and AQR, PQR, URX, respectively.

a - c *flp-8* and *flp-21* are strongly and relatively specifically expressed in AQR, PQR and/or URX neurons. Data taken from Taylor *et al.* (Taylor, S. R. et al. Molecular topography of an entire nervous system. Cell 184, 4329-4347.e23 (2021)) that were obtained for N2 wild-type worms at the L4 stage.

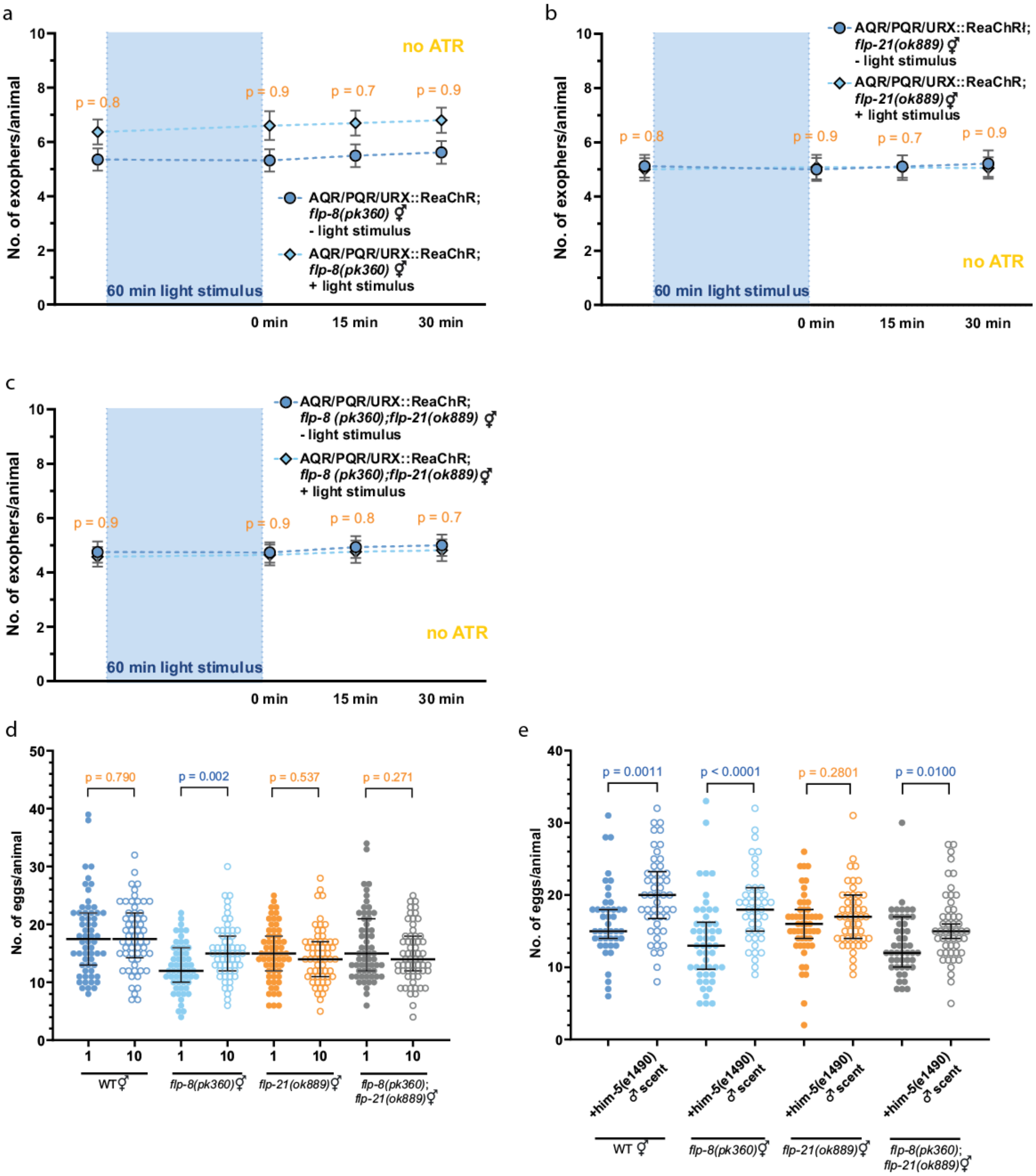

Supplementary Figure 7. Control experiments without all-trans retinal (ATR) ReaChR-mediated activation of AQR, PQR, and URX neurons that do not produce FLP-8 and/or FLP-21 neuropeptides.

a Control experiment without all-trans retinal (ATR) ReaChR-mediated activation of AQR, PQR, and URX neurons in *flp-8(pk360)* mutant background. n = 57 worms (-light stimulus), and 58 worms (+ light stimulus), N = 5 independent experiments.

b Control experiment without all-trans retinal (ATR) ReaChR-mediated activation of AQR, PQR, and URX neurons in *flp-21(ok889)* mutant background. n = 57 worms (-light stimulus), and 58 worms (+ light stimulus), N = 5 independent experiments.

c Control experiment without all-trans retinal (ATR) ReaChR-mediated activation of AQR, PQR, and URX neurons in *flp-8(pk360), flp-21(ok889)* double mutant background.

n = 60 worms (-light stimulus), and 59 worms (+ light stimulus), N = 5 independent experiments.

d *In utero* embryo number for wild-type worms, *flp-8(pk360)* and *flp-21(ok889)* single and double mutants grown as solitary animals or in ten-hermaphrodite population. n = 60, 60, 55, 59, 60, 60, 59, and 60 worms (for respective columns), N = 3 independent experiments.

e *In utero* embryo number for wild-type worms, *flp-8(pk360)* and *flp-21(ok889)* single and double mutants grown on male-conditioned plates. n = 43, 50, 50, 43, 51, 50, 47, and 50 worms (for respective columns), N = 2 independent experiments.

Data information: +ATR means “with all-trans-retinal”; no ATR means “without all-trans-retinal”. Data are presented as mean with SEM (a - c) or as median with interquartile range (d - e); not significant p values ($p > 0.05$) are in orange color, significant p values ($p < 0.05$) are in blue color; two-tailed Mann-Whitney test. Source data are provided as a Source Data file.

Supplementary Tables

Supplementary Table 1. Differential expression analysis of detected GPCRs. Bold font indicates the name of the transcript analyzed in detail in the manuscript. The two-sided Wald test was used to generate pvalues and log2FoldChanges. Padj represents the adjusted p-value using the Benjamini & Hochberg method.

Gene.name	log2FoldChange	pvalue	padj
srw-86	2.470005259	0.006089231	NA
str-173	**1.210254202**	**0.083872612**	**NA**
sri-39	0.961870706	0.118312493	NA
srm-1	0.74631012	0.197705915	NA
srh-269	0.715357507	0.20128121	NA
sri-36	0.697436016	0.069365061	NA
str-55	0.69275127	0.286227955	NA
srd-64	0.671778641	0.227938718	NA
srsx-36	0.632246484	0.436691291	NA
sri-57	0.609273781	0.193809518	NA
srw-89	0.578848267	0.407242411	NA
srbc-82	0.56597091	0.423856002	NA
srr-2	0.523068614	0.103943944	NA
srx-97	0.505797282	0.424229255	NA
sri-11	0.489747472	0.297473585	NA

srv-15	0.463355568	0.372243301	NA
srj-14	0.460503239	0.065003699	NA
sre-36	0.458443613	0.346744567	NA
srh-217	0.429350684	0.444951979	NA
srh-2	0.409544299	0.241263936	NA
srx-41	0.39088958	0.531069383	NA
srr-10	0.382508507	0.600239609	NA
srz-97	0.370702424	0.498470233	NA
srsx-19	0.353102397	0.54270153	NA
srd-70	0.320458039	0.541451057	NA
srh-71	0.308999771	0.434944825	NA
str-179	0.291901118	0.596460956	NA
str-148	0.267146531	0.651806161	NA
sra-35	0.258242663	0.576292957	NA
srx-45	0.226491874	0.636877507	NA
srsx-27	0.225810039	0.724940673	NA
srr-6	0.223992203	0.491120033	NA
srb-16	0.213930895	0.686266407	NA
sri-55	0.204504014	0.648957813	NA
srx-125	0.196087663	0.632165424	NA

srw-48	0.1760458	0.784611201	NA
sri-35	0.174186697	0.816308334	NA
srsx-25	0.172603337	0.699023544	NA
srj-29	0.145173827	0.82575966	NA
str-176	0.145080793	0.381737839	0.705706926
srv-32	0.140744356	0.836820773	NA
srj-52	0.12758145	0.8360209	NA
srab-4	0.123433522	0.75301419	NA
srxa-14	0.122166676	0.825629763	NA
src-1	0.121226527	0.017785109	0.153059765
srr-4	0.118248088	0.143496801	0.45832863
srsx-34	0.118146738	0.517657949	NA
str-144	0.115566741	0.882522042	NA
sri-8	0.114856655	0.747072742	NA
srh-48	0.107505544	0.732564142	NA
str-245	0.085385814	0.731316789	NA
srv-7	0.081868619	0.676931931	NA
srj-9	0.057842686	0.909552581	NA
sra-33	0.04490344	0.924342693	NA
srsx-18	0.037063376	0.9551973	NA

src-2	0.006822308	0.971252278	NA
srr-1	0.00459541	0.99125387	NA
sri-59	0.000405882	0.999217048	NA
srg-34	-0.030080578	0.947775572	NA
str-172	-0.085472421	0.87286706	NA
srr-3	-0.088516378	0.76868265	NA
srd-32	-0.107551827	0.816267341	NA
srv-1	-0.11334895	0.410156047	0.727497177
srh-237	-0.151085831	0.175026351	0.503664735
str-183	-0.178110179	0.787945578	NA
sra-10	-0.178642194	0.779315606	NA
sra-32	-0.190091468	0.685526539	NA
srd-14	-0.207486411	0.305360875	NA
sre-13	-0.210537797	0.702655411	NA
srd-52	-0.235477439	0.677464298	NA
sri-40	-0.247157736	0.037111528	0.232197335
srz-85	-0.247831631	0.640803343	NA
sra-14	-0.266593158	0.200655026	NA
srh-16	-0.275054658	0.57204149	NA
srh-70	-0.322126344	0.575669232	NA

srab-14	-0.328373116	0.605637275	NA
srx-12	-0.361291214	0.429781376	NA
sre-9	-0.397855081	0.275566108	NA
srx-58	-0.458521562	0.487282023	NA
sri-5	-0.458897325	0.308597251	NA
srt-23	-0.460153207	0.606235802	NA
srm-3	-0.470085367	0.174616055	NA
srh-33	-0.479357546	0.48607886	NA
srh-105	-0.486191119	0.439431381	NA
srw-35	-0.492290252	0.480189518	NA
srx-118	-0.521178132	0.380103143	NA
srm-5	-0.557246057	0.448970879	NA
srd-53	-0.588697815	0.325322641	NA
str-168	-0.611511779	0.293797037	NA
sre-4	-0.659337898	0.279814818	NA
srx-98	-0.789769153	0.126804165	NA
srt-39	-0.803397597	0.204138417	NA
srz-99	-0.866876045	0.048915705	NA
sre-44	-1.105518367	0.091985454	NA

Supplementary Table 2. List of all exopher-related phenotypes presented in the manuscript.

Figure no.	Condition	Observed phenotype
1c	Exopher level in hermaphrodites in the presence of *him-5(e1490)* males	Increased exopher number in *him-5(e1490)* mutant hermaphrodites
1d	*In utero* embryo retention in the presence of *him-5(e1490)* males	Increased embryo retention in *him-5(e1490)* mutant hermaphrodites
1f	Effect on exopher production in hermaphrodites grown on WT and *him-5* males-conditioned plates	Increased exopher number after exposure to *him-5* and WT males secretome
1g	Effect on *in utero* embryo retention in hermaphrodites grown on WT and *him-5* males-conditioned plates	Increased *in utero* embryo retention after exposure to *him-5* and WT males secretome
1i	Effect of male pheromones presence on exopher production in different developmental time frames	Increased exopher number (strongest effect on exposure during L4 to AD1 stages)
1j	Effect of male pheromones presence on *in utero* embryo retention in different developmental time frames effect	Increased *in utero* embryo retention (apart from exposure during L4 to AD1 stages)
2b	Population size effect on exopher production (single or 10 hermaphrodites)	Single worms produce more exophers than worms cultured in 10 hermaphrodite population
2c	Population size effect on *in utero* embryo retention	No effect
2e	Population size effect on exopher production (single, 5, or 10 hermaphrodites)	Exopher number decreased in 5 and 10 hermaphrodites populations
2f	Population size effect on exopher production (single, 10, or 100 hermaphrodites)	Exopher number decreased in 10 and 100 hermaphrodite populations
2g	Hermaphrodites-conditioned plates effect on exopher production	Hermaphrodite pheromones similarly decrease exopher number as hermaphrodite presence

2h	Larvae presence effect on exopher production	Larvae presence decreases exopher production
3a	Effect of ascr#3 exposure from L4 to AD2 on exophergenesis	Decrease in exopher number after exposure to 1pg or 1ng of ascr#3
3b	Effect of ascr#3 exposure from embryo to AD2 on exophergenesis	No effect
3c	Effect of ascr#10 exposure from L4 to AD2 on exophergenesis	Dose-dependent effect– increase in exopher production after 1ng ascr#10 exposure; exposure to 1 pg ascr#10 has no effect
3d	Effect of ascr#10 exposure from embryo to AD2 on exophergenesis	Dose-dependent effect– increase in exopher production after 1ng ascr#10 exposure; exposure to 1 pg ascr#10 has no effect
3g	Influence of ascaroside side-chain biosynthesis mutants' presence on exophergenesis level in wild-type worms	Number of exophers elevated in the presence of *acox-1* mutants; decreased exopher level in the presence of *maoc-1* mutants; *daf-22* mutants presence does not affect exophergenesis
3h	Influence of ascaroside side-chain biosynthesis mutants' presence on *in utero* embryo retention	No influence
4b	Influence of impaired ciliated sensory neurons on exophergenesis in *che-13* mutant	*che-13* mutant produces fewer exophers than wild-type worms
4c	Genetic ablation of chemosensory neurons effect on exopher production	ASK(-), AWC(-), ADL(-), or ASH(-) mutants produce fewer exophers
4d	Genetic ablation of chemosensory neurons effect on *in utero* embryo retention	ASK(-), AWC(-), ADL(-), ASH(-) and CEM(+) have decreased *in utero* embryo retention
4e	Male-specific pheromones effect on exophergenesis in mutants with ablated chemosensory neurons	ASK(-), ADL(-), AWB(-) – no effect<br>WT, AWC(-), ASH(-), ASI(-), CEM(+) – increased exophergenesis after male pheromones exposure
4f	Population size effect on exopher production in mutants with ablated chemosensory neurons	ASH(-), ASI(-), CEM(-), ASK(-) – no effect<br>AWC(-), ADL(-), AWB(-) – exopher number decreased in worms grown in the population of 10 hermaphrodites

5a	*str-173* expression visualization in L1, L4 and AD2 worms	*str-173* is expressed in ASK neurons, vulva, and the tail in L4 and adult worms;<br>L1 larvae express *str-173* in pharynx
5b	*str-173* expression visualization in L4 worms	*str-173* is expressed in ASK neurons
5c	Exopher level in *str-173* mutants	Exopher production is not altered in *str-173* mutants
5d	Population size effect on exopher production in *str-173* mutants	*str-173(wwa1)* mutation does not affect exopher production in worms grown in separation or in 10 hermaphrodites population; *str-173(wwa2)* – higher exopher level in worms grown in separation
5e	Male-specific pheromones effect on exopher production in *str-173* mutants	No effect
5f	Effect of ascr#10 exposure on exophergenesis in *str-173* mutants	Decrease in exopher production
5g	Effect of ascr#10 on eggs in utero retention in *str-173* mutants	Decreased *in utero* embryo retention
5h	Effect of ascr#10 exposure from embryo to AD2 on exophergenesis in ASK(-) mutant	No effect
5i	Effect of ascr#3 exposure from embryo to AD2 on exophergenesis in ASK(-) mutant	Decrease in exopher production
6a	Ablation of AQR/PQR/URX neurons effect on exopher level	Increased exopher number in AQR/PQR/URX(-) mutant
6b	Ablation of AQR/PQR/URX neurons affect *in utero* embryo retention	Decreased *in utero* embryo retention in AQR/PQR/URX(-) mutant
6c	ArchT-mediated AQR/PQR/URX<br>inactivation effect on exopher production (AD2)	Increased exopher number after AQR/PQR/URX inactivation
6d	ReaChR-mediated AQR/PQR/URX activation effect on exopher production (AD2)	Decreased exopher number after AQR/PQR/URX activation

6e	Ablation of ASK/AQR/PQR/URX neurons effect on exopher level	Low exophergenesis levels in animals with genetic ablation of ASK neurons and high levels of exophergenesis in animals with genetic ablation of AQR, PQR, and URX neurons are equalized in animals with all four neurons removed
6f	Population size effect on exopher production in AQR/PQR/URX(-) mutant	No effect
6g	Male-specific pheromones effect on exopher production in AQR/PQR/URX(-) mutant	Increase in exopher production after exposure to male secretome
6h	FUdR effect on exophergenesis in AQR/PQR/URX(-) mutant	Decreased exopher production in aminals treated with FUdR is not rescued by AQR/PQR/URX removal
6i	*rme-2* knockdown effect on exophergenesis in AQR/PQR/URX(-) mutant	AQR/PQR/URX removal only partially rescues the inhibition of exophergenesis caused by *rme-2* knockdown
7a	Exopher level in *flp-8(pk360)* and *flp-21(ok889)* mutants	Increased exopher production in single *flp-8* and *flp-21* mutants, and in *flp-8; flp-21* double mutant
7b	Number of *in utero* embryos in *flp-8(pk360)* and *flp-21(ok889)* mutants	No change
7c	ReaChR-mediated AQR/PQR/URX; *flp-8(pk360)*activation effect on exopher production (AD2)	Increased exopher production
7d	ReaChR-mediated AQR/PQR/URX; *flp-21(ok889)* activation effect on exopher production (AD2)	No effect
7e	ReaChR-mediated AQR/PQR/URX; *flp-8(pk360)*; *flp-21(ok889)* activation effect on exopher production (AD2)	No effect
7f	Population size effect on exopher production in *flp-8(pk360), flp-*	No effect

	21(ok889), and *flp-8(pk360)/flp-21(ok889)* mutants	
7g	Male-specific pheromones effect on exopher production in *flp-8(pk360), flp-21(ok889),* and *flp-8(pk360)/flp-21(ok889)* mutants	Increased exopher production
Sup. Fig. 1a	Males-conditioned plates seeded with HT115 bacteria strain effect on exopher production	Increase in exopher production
Sup. Fig. 1c	*him-5* males secretions effect on *him-5* hermaphrodites' exopher production	No effect
Sup. Fig. 1d	Co-culturing *him-5* hermaphrodites with *him-5* males	Increased exopher production
Sup. Fig. 2a	Effect of ascr#3 on *in utero* embryo retention	No effect
Sup. Fig. 2b	Effect of ascr#10 on *in utero* embryo retention	No effect
Sup. Fig. 3a	Influence of single neuron class removal on exopher production in solitary animals	ASK(-), AWC(-), ADL(-), ASH(-), CEM(+) – decreased exopher production<br>AWB(-), ASI(-) – no change
Sup. Fig. 3b	Influence of AWC neuron absence on exophergenesis in different temperatures	AWC neurons regulate the temperature-dependent increase in muscle exopher production
Sup. Fig. 3c	Male-specific pheromones effect on egg retention in mutants with ablated chemosensory neurons	ASI(-) – increased number of *in utero* embryos after exposure to male pheromones<br>ASK(-), ADL(-), AWB(-) – no change
Sup. Fig. 3d	Population size effect on *in utero* embryos retention in mutants with ablated chemosensory neurons	ASI(-) -more embryos retained in worms grown in the population of 10 hermaphrodites<br>AWB(-) – no change
Sup. Fig. 3e	Influence of ascaroside side-chain biosynthesis mutants presence on exophergenesis level in ASI(-) mutants	No influence

Sup. Fig. 3f	Influence of ascaroside side-chain biosynthesis mutants presence on exophergenesis level in AWB(-) mutants	No influence
Sup. Fig. 4a	Population size effect on the number of exophers produced by worms that were used for RNAseq analysis	Worms cultured in a population of 10 hermaphrodites – fewer exophers produced in each biological repeat
Sup. Fig. 5a	Number of eggs laid by AQR/PQR/URX(-) mutant	Decreased number of laid eggs by AQR/PQR/URX(-) mutant
Sup. Fig. 5b	Number of eggs laid by AQR/PQR/URX(-) mutant on consecutive days of adulthood	No significant change
Sup. Fig. 5c	ArchT-mediated AQR/PQR/URX inactivation effect on exopher production (no ATR control)	No change
Sup. Fig. 5d	ReaChR-mediated AQR/PQR/URX activation effect on exopher production (no ATR control)	No change
Sup. Fig. 5e	ArchT-mediated AQR/PQR/URX inactivation effect on exopher production (AD1)	No change
Sup. Fig. 5f	ArchT-mediated AQR/PQR/URX inactivation effect on exopher production (no ATR control; AD1)	No change
Sup. Fig. 5g	ReaChR-mediated AQR/PQR/URX activation effect on exopher production (AD1)	Decrease in exopher production after 30 min light stimulation
Sup. Fig. 5h	ReaChR-mediated AQR/PQR/URX activation effect on exopher production (no ATR control, AD1)	No change
Sup. Fig. 7a	ReaChR-mediated AQR/PQR/URX; *flp-8(pk360)* activation effect on exopher production (no ATR control)	No change

Sup. Fig. 7b	ReaChR-mediated AQR/PQR/URX; *flp-21(ok889)* activation effect on exopher production (no ATR control)	No change
Sup. Fig. 7c	ReaChR-mediated AQR/PQR/URX; *flp-8(pk360)*; *flp-21(ok889)*<br><br>activation effect on exopher production (no ATR control)	No change
Sup. Fig. 7d	Population size effect on *in utero* embryos retention in *flp-8(pk360)*, *flp-21(ok889)*, and *flp-8(pk360)/flp-21(ok889)* mutants	*flp-21*, *flp-8/flp-21* – no effect<br><br>*flp-8* – increased in utero embryos retention
Sup. Fig. 7e	Effect on *in utero* embryo retention in *flp-8(pk360)*, *flp-21(ok889)*, and *flp-8(pk360)/flp-21(ok889)* mutant hermaphrodites grown on *him-5* males-conditioned plates	*flp-8*, *flp-8/flp-21* – increased in utero embryos retention<br><br>*flp-21* – no change

Description of Supplementary Data Files

File name: Supplementary Data 1

Description: List of reagents and software used in the study.

File name: Supplementary Data 2

Description: List of *Caenorhabditis elegans* strains used in the study.

File name: Supplementary Data 3

Description: List of recombinant DNA and oligonucleotides used in the study.
