# Supplementary material for: Pheromone-based communication influences the production of somatic extracellular vesicles in C. elegans
Source: Nat Commun. 2024 Mar 28;15:2715. doi: 10.1038/s41467-024-47016-x (PMC10978837; doi:10.1038/s41467-024-47016-x)
Supplement: Supplementary file 3 — Description of Additional Supplementary Files [file 41467_2024_47016_MOESM3_ESM.pdf]

Description of Additional Supplementary Files

File name: Supplementary Data 1

Description: List of reagents and software used in the study.

File name: Supplementary Data 2

Description: List of *Caenorhabditis elegans* strains used in the study.

File name: Supplementary Data 3

Description: List of recombinant DNA and oligonucleotides used in the study.
