# Supplementary material for: Pheromone-based communication influences the production of somatic extracellular vesicles in C. elegans
Source: Nat Commun. 2024 Mar 28;15:2715. doi: 10.1038/s41467-024-47016-x (PMC10978837; doi:10.1038/s41467-024-47016-x)
Supplement: Supplementary file 7 — Reporting Summary [file 41467_2024_47016_MOESM7_ESM.pdf]

nature portfolio

Corresponding author(s): Michał Turek
Wojciech Pokrzywa

Last updated by author(s): Feb 28, 2024

Reporting Summary

Nature Portfolio wishes to improve the reproducibility of the work that we publish. This form provides structure for consistency and transparency in reporting. For further information on Nature Portfolio policies, see our Editorial Policies and the Editorial Policy Checklist.

Statistics

For all statistical analyses, confirm that the following items are present in the figure legend, table legend, main text, or Methods section.

n/a	Confirmed	
☐	☒	The exact sample size (*n*) for each experimental group/condition, given as a discrete number and unit of measurement
☐	☒	A statement on whether measurements were taken from distinct samples or whether the same sample was measured repeatedly
☐	☒	The statistical test(s) used AND whether they are one- or two-sided<br>*Only common tests should be described solely by name; describe more complex techniques in the Methods section.*
☒	☐	A description of all covariates tested
☐	☒	A description of any assumptions or corrections, such as tests of normality and adjustment for multiple comparisons
☐	☒	A full description of the statistical parameters including central tendency (e.g. means) or other basic estimates (e.g. regression coefficient) AND variation (e.g. standard deviation) or associated estimates of uncertainty (e.g. confidence intervals)
☐	☒	For null hypothesis testing, the test statistic (e.g. *F*, *t*, *r*) with confidence intervals, effect sizes, degrees of freedom and *P* value noted<br>*Give P values as exact values whenever suitable.*
☒	☐	For Bayesian analysis, information on the choice of priors and Markov chain Monte Carlo settings
☒	☐	For hierarchical and complex designs, identification of the appropriate level for tests and full reporting of outcomes
☒	☐	Estimates of effect sizes (e.g. Cohen's *d*, Pearson's *r*), indicating how they were calculated

Our web collection on statistics for biologists contains articles on many of the points above.

Software and code

Policy information about availability of computer code

Data collection	Confocal images were taken using Zen 2012 SP5 FP3 software (Zeiss)
Data analysis	Data were analysed using GraphPad Prism 9 (GraphPad Software, Inc.), Office 365 (Microsoft), and ImageJ (Wayne Rasband). Data were visualised using GraphPad Prism 9 (GraphPad Software, Inc.), BioRender (Biorender.com), and Adobe Illustrator CS6 (Adobe).

For manuscripts utilizing custom algorithms or software that are central to the research but not yet described in published literature, software must be made available to editors and reviewers. We strongly encourage code deposition in a community repository (e.g. GitHub). See the Nature Portfolio guidelines for submitting code & software for further information.

Data

Policy information about availability of data

All manuscripts must include a data availability statement. This statement should provide the following information, where applicable:

- Accession codes, unique identifiers, or web links for publicly available datasets
- A description of any restrictions on data availability
- For clinical datasets or third party data, please ensure that the statement adheres to our policy

The authors declare that the main data supporting the findings of this study are available within the article and its supplementary files. Source data are provided with this paper. The RNAseq data generated in this study have been deposited in the GEO database under accession code GSE241786 and can be accessed using the

following link: https://www.ncbi.nlm.nih.gov/geo/query/acc.cgi?acc=GSE241786. All raw data have been deposited at Zenodo repository and can be accessed at https://zenodo.org/doi/10.5281/zenodo.10718455.

Research involving human participants, their data, or biological material

Policy information about studies with human participants or human data. See also policy information about sex, gender (identity/presentation), and sexual orientation and race, ethnicity and racism.

Reporting on sex and gender	N.A.
Reporting on race, ethnicity, or other socially relevant groupings	N.A.
Population characteristics	N.A.
Recruitment	N.A.
Ethics oversight	N.A.

Note that full information on the approval of the study protocol must also be provided in the manuscript.

Field-specific reporting

Please select the one below that is the best fit for your research. If you are not sure, read the appropriate sections before making your selection.

☒ Life sciences ☐ Behavioural & social sciences ☐ Ecological, evolutionary & environmental sciences

For a reference copy of the document with all sections, see nature.com/documents/nr-reporting-summary-flat.pdf

Life sciences study design

All studies must disclose on these points even when the disclosure is negative.

Sample size	No statistical method were used to predetermine sample size. Sample sizes are similar to those used in the field (as previously reported: Turek et al. EMBO Reports 2020) and were done accordingly to standard C. elegans approaches. Exact sample sizes are indicated in the corresponding figure legends.
Data exclusions	No data were excluded from the analysis.
Replication	Each experiment was repeted at least three times (except for three which were repeated two times) in independent experiments in multiple animals. Experimental findings were reproduced reliably.
Randomization	Worms were randomly allocated to the experimental groups for all the data sets.
Blinding	Investigators were blinded to group allocation during data collection and analysis for most of the experiments performed within this study. A list of blinded experiments is presented in the Methods section. Blinding was not performed only for these experiments which were performed by the same investigator throughout the whole procedure and because of the unambiguous nature of the measurements and systematic analysis used in these experiments. However, the different conditions were assessed in random order.

Reporting for specific materials, systems and methods

We require information from authors about some types of materials, experimental systems and methods used in many studies. Here, indicate whether each material, system or method listed is relevant to your study. If you are not sure if a list item applies to your research, read the appropriate section before selecting a response.

Materials & experimental systems

n/a	Involved in the study	
☒	☐	Antibodies
☒	☐	Eukaryotic cell lines
☒	☐	Palaeontology and archaeology
☐	☒	Animals and other organisms
☒	☐	Clinical data
☒	☐	Dual use research of concern
☒	☐	Plants

Methods

n/a	Involved in the study	
☒	☐	ChIP-seq
☒	☐	Flow cytometry
☒	☐	MRI-based neuroimaging

Animals and other research organisms

Policy information about studies involving animals; ARRIVE guidelines recommended for reporting animal research, and Sex and Gender in Research

Laboratory animals	In this study, we used the nematode Caenorhabditis elegans. Experiments were performed using worms at adulthood day 2. C. elegans strains: N2 (wild type), ACH81, ACH93, RB859, WOP456, VC1785, TUR7, RB2000, TUR46, TUR11, TUR16, TUR75, WOP499, TUR49, TuR47, TUR51, TUR18, TUR37, TUR59, TUR38, TUR43, PHX6501, TUR77, TUR61, TUR62, WOP558, WOP559, TUR84, TUR85, TUR93, TUR94, TUR97, TUR96.
Wild animals	The study did not involve wild animals.
Reporting on sex	The study was performed on hermaphrodites as tin C. elegans exophergenesis occurs only in hermaphrodites.
Field-collected samples	The study did not involve samples collected from the field.
Ethics oversight	Studies on the nematode Caenorhabdities elegans require no ethical approval.

Note that full information on the approval of the study protocol must also be provided in the manuscript.
